# Nuclear Localization of Effector BPE159: A Pivotal Mechanism for Intracellular Persistence of *Brucella* by Hampering Host Autophagy

**DOI:** 10.3390/microorganisms14030663

**Published:** 2026-03-14

**Authors:** Yidan Zhang, Tingting Lyu, Shengnan Song, Yu Zhang, Chunyan Wei, Liangbo Liu, Zhen Wang, Zhihua Sun, Xia Zhou, Jia Guo, Hui Zhang

**Affiliations:** State International Joint Research Center for Animal Health Breeding, College of Animal Science and Technology, Shihezi University, Shihezi 832003, China; zyd19990866698@163.com (Y.Z.); 20232013041@stu.shzu.edu.cn (T.L.); songshengnan0123@126.com (S.S.); 18997730217@163.com (Y.Z.); 18703098523@163.com (C.W.); llb@shzu.edu.cn (L.L.); wzhen2018@shzu.edu.cn (Z.W.); zhihuasun918@shzu.edu.cn (Z.S.); zhoux993@163.com (X.Z.)

**Keywords:** *Brucella abortus*, secreted proteins, autophagy

## Abstract

*Brucella* is a neglected foodborne pathogen, which contaminates milk, dairy products, meat, and meat products of infected animals. However, the role of the *Brucella* putative effector (BPE) protein family, which relies on the type IV secretion system (T4SS) in *Brucella abortus*, remains unclear. We demonstrated that BPE159 mediates the regulation of host nuclei in autophagy. The host-interacting protein Eci1 was screened using yeast two-hybridization, molecular docking, and immunoprecipitation, and *BPE159*-deleted (ΔBPE159) and complementary (ΔBPE159-C) strains were constructed by homologous recombination. We evaluated their growth, survival, and replication and measured the expression of autophagy-related cytokine mRNAs in macrophages. BPE159 was localized in the nucleus of host cells and interacted with Eci1 to downregulate the expression of macrophage autophagy factors, thereby inhibiting host autophagy and enabling the persistence of *Brucella*. These findings highlight the critical role of BPE159 in mediating autophagy through Eci1 in host cells to promote *Brucella* survival in host cells.

## 1. Introduction

*Brucella* is an important bacterial pathogen affecting humans and animals. Brucellosis can be transmitted to humans through the consumption of contaminated food and dairy products, occupational exposure, or the inhalation of infected aerosols [1]. Brucellosis in livestock can lead to clinical signs such as miscarriage, infertility, joint swelling, orchitis, and decreased milk production. As a parasitic bacterium within the envelope, when *Brucella* invades host cells, it forms special *Brucella*-containing vacuole (BCV) structures for evading lysosomal clearance. BCVs that acquire endosomal properties are called endosomal *Brucella*-containing vacuoles (eBCVs) [2]. Immediately afterward, eBCVs accumulate with the endoplasmic reticulum (ER)-derived membrane through eBCV-membrane reversal and interact with the ER to obtain its structural and functional characteristics [3], ultimately becoming replicative BCVs (rBCVs). Finally, owing to the proliferation of *Brucella* and eventual formation of rBCVs, called autophagocytic BCVs, with late lysosomal characteristics [2], the intracellular circulation process of *Brucella* is completed, and mature *Brucella* is released.

In this process, the type IV secretion system (T4SS) regulates the maturation and intracellular transport of BCV by secreting various effector proteins and helps to evade immune recognition by the host body, thereby maintaining long-term survival and persistent infection of *Brucella* within the host. Currently, 16 T4SS effector proteins are known [4]. Among them, the VirB T4SS system expresses and secretes VceC—which binds to the ER chaperone Grp78/BiP—which activates the unfolded protein response and ultimately triggers an inflammatory response [5]. BtpA and BtpB are homologs of the eukaryotic Toll/Interleukin-1 receptor domain. They modulate immune responses in host cells by directly disrupting Toll-like receptor signaling pathways via their Toll/Interleukin-1 receptor domains [6].

Nuclear regulatory proteins affect transcription and other biological processes by intervening in the host cell nucleus. Secreted effector proteins and transport nucleoprotein complexes are one of the important ways for some intracellular bacteria to show virulence [7,8]. *Brucella* putative effector (BPE) protein family, mainly including BPE865, BPE159, BPE123, BPE005, BPE043, and BPE275—secreted as proposed by the CyaA reporting system—plays an important role as virulence factors by mediating the stability of the bacterial outer membrane, promoting intracellular transport, coordinating the expression of other virulence factors for affecting *Brucella* survival, regulating host inflammatory responses, and participating in bacterial nutrient acquisition and metabolic regulation. For example, BPE123 inhibits autophagy by interacting with the host target protein IFT20, and it can target α-enolase in host cells, thereby prolonging the survival time of *Brucella* in cells [9]. However, the biological function of secreted BPE159 and underlying molecular mechanism remain unclear.

*Eci1*, also known as mouse enoyl-CoA δ isomerase 1, is located on chromosome 16 and is a nuclear gene of mitochondrial products. Eci1 is expressed in various tissues and organs, specifically the kidneys and colon.

We identified BPE159 as a nuclear protein. We screened three proteins interacting with BPE159 (Eif3g, Mrpl19, and Eci1) and experimentally confirmed that the host protein Eci1 binds to BPE159. When BPE159 is absent, Eci1 transcriptional level significantly decreases. This reduction promotes the expression of autophagy factors, thereby hindering the intracellular survival of *Brucella*.

## 2. Materials and Methods

### 2.1. Bacterial Strains and Cells

*Brucella abortus* wild-type strain (S2308) was provided by the Chinese Center for Disease Control and Prevention (Beijing, China). The strains were cultured in tryptic soy broth or on tryptic soy agar (TSA) (Difco, Franklin Lakes, NJ, USA) plates at 37 °C under 5% CO_2_. *E. coli* DH5α and yeast AH109 (MATα) (Clontech, Mountain View, CA, USA) were cultured in Luria–Bertani broth and on synthetic dropout (SD) plates, respectively. RAW264.7 mouse macrophage and HEK293T human embryonic kidney cell lines were provided by the Key Laboratory of Animal Disease Prevention and Control of Shihezi University (Xinjiang, China) and cultured in Dulbecco’s Modified Eagle Medium (Thermo Fisher Scientific, Waltham, MA, USA) containing 10% fetal bovine serum (Gibco, Carlsbad, UK) at 37 °C under 5% CO_2_.

### 2.2. Bioinformatic Analysis of BPE159

*BPE159* sequence of *B. abortus* S2308 (BAB1_0159) (GenBank accession number: NC_007618.1) was obtained from the NCBI GenBank database. ExPASy ProtParam (https://web.expasy.org/protparam/ (accessed on 1 November 2025)) was used to predict the number of amino acids, coefficient of instability, molecular weight, total number of atoms, theoretical isoelectric point, total average hydrophobicity, and other relevant physicochemical parameters of BPE159. The hydrophobicity of BPE159 was predicted using the ExPASy ProtScale tool (https://web.expasy.org/protscale/ (accessed on 1 November 2025)). TMHMM (http://www.cbs.dtu.dk/services/TMHMM/ (accessed on 1 November 2025)) and online analysis software were used to identify the transmembrane domain of BPE159. Epitopes of BPE159 were predicted using PREDICTED ANTIGENIC PEPTIDES (http://imed.med.ucm.es/Tools/antigenic.pl (accessed on 1 November 2025)). The secondary structure of BPE159 was predicted using SOPMA (https://npsa.lyon.inserm.fr/ (accessed on 1 November 2025)). The biological prediction software cNLSMapper (https://nls-mapper.iab.keio.ac.jp/ (accessed on 1 November 2025)) was used to analyze the nuclear localization signal of BPE159. RaptorX (http://raptorx.uchicago.edu (accessed on 1 November 2025)) was used to analyze the tertiary structure of BPE159. The potential functions of interacting proteins and their corresponding signaling pathways were analyzed using String software (https://string-db.org/cgi/input.pl (accessed on 1 November 2025)), Gene Ontology (GO) database, and Kyoto Encyclopedia of Genes and Genomes (KEGG) database. Molecular docking was performed via the Easy2MD online platform (https://easy2md.com/ (accessed on 1 November 2025)).

### 2.3. Amplification of BPE159 and Plasmid Construction

*BPE159* was amplified and the polymerase chain reaction (PCR) products were cloned into pMD19-T. The plasmid was then cloned into the yeast expression vector pGBKT7 (Ouyi Biotechnology, Shanghai, China) and eukaryotic expression vectors pDsRed2-C1, pCDNA3.1-EGFP, pCMV-HA, and pAcGFP1-C1 using EcoRI and XhoI to obtain pGBKT7-BPE159, pDsRed2-C1-BPE159, pCMV-HA-BPE159, pAcGFP1-C1-Eci1, and pCDNA3.1-EGFP-Eci1, respectively.

### 2.4. Primer Designing

Based on the *BPE159* sequence deposited in GenBank (NC_007618.1), specific primers were designed using Primer v.5.0 (Supplementary Table S1). These primers were used to construct the *BPE159* deletion and complementation strains and construction of fluorescent co-localization vectors. Additionally, they were used to perform quantitative reverse-transcription polymerase chain reaction (qRT-PCR).

### 2.5. Determination of Cellular Localization of BPE159 by Laser Confocal Microscopy

HEK293T cells were seeded onto glass coverslips placed on a 24-well plate. Upon reaching approximately 70% confluency, the cells were transfected with the plasmid pDsRed2-C1-BPE159, either alone or in combination with the pCDNA3.1-EGFP-Eci1 vector using Lipo8000 transfection reagent (Invitrogen, Waltham, MA, USA). After 24 h of transfection, the cells were rinsed with phosphate-buffered saline (PBS), and the nuclei were counterstained with DAPI (Solarbio, Beijing, China) for 5 min in the dark. Following staining, an antifade mounting medium (Solarbio, China) was applied to the samples. The subcellular localization of the BPE159 protein was then visualized and analyzed using a confocal laser scanning microscope (Nikon C2i+, Tokyo, Japan) [10].

### 2.6. Yeast Two-Hybrid Screening for BPE159-Interacting Proteins

The pGBKT7-BPE159 plasmid was introduced into the *Saccharomyces cerevisiae* strain AH109 (MATα; Clontech, USA) to assess the potential self-activation and cytotoxicity of BPE159. Transformants were first selected on SD medium lacking leucine and tryptophan SD/–Leu/–Trp (DDO) plates (Coolaber, Beijing, China). Single colonies (>2 mm in diameter) were inoculated into DDO liquid medium and grown to the exponential phase under shaking (30 °C, 200 rpm, 200 h). Cultures were then plated onto more stringent SD medium lacking adenine, histidine, leucine, and tryptophan SD/–Ade/–His/–Leu/–Trp (QDO) plates (Coolaber, China). Sterile spots on DDO and QDO plates indicate that the BPE159 protein is toxic. Spots on DDO plates indicate that the protein has self-activation activity. White spots on DDO plates and sterile spots on QDO plates indicate that the protein does not have self-activation activity.

For yeast two-hybrid screening, the pGBKT7-BPE159 construct was introduced into AH109 cells via the PEG/LiAc transformation method, with salmon sperm DNA (Coolaber, China) used as carrier DNA during denaturation. Subsequently, the pGADT7-cDNA library was co-transformed into the same strain. Positive interactions were screened sequentially on DDO, QDO, and SD/–Ade/–His/–Leu/–Trp/X-α-gal (QDO/X) plates (Coolaber, China). Candidate clones obtained from the QDO/X plates were sequenced and analyzed using the BLAST (Basic Local Alignment Search Tool) (https://blast.ncbi.nlm.nih.gov (accessed on 1 November 2025)) algorithm for identity confirmation.

The plasmids from positive clones were extracted using a yeast plasmid extraction kit (Solarbio, China) and subsequently transformed into *E. coli* DH5α competent cells (Clontech, USA). Following culture expansion, the plasmid was purified with a plasmid extraction kit (TIANGEN, Beijing, China). The purified plasmid, together with pGBKT7-BPE159, was then co-transformed into *S. cerevisiae* strain AH109. Transformants were screened on selective media: DDO, QDO, and QDO/X plates [11].

### 2.7. Co-Immunoprecipitation Experiments

The plasmids pCMV-HA-BPE159 and pAcGFP1-C-Eci1 were purified using an endotoxin-free plasmid miniprep kit (TIANGEN, China) and co-transfected into HEK293T cells. After 48 h, cells were lysed with 1 mL of IP lysis buffer (Thermo Fisher Scientific, USA), and total protein extracts were collected for Western blot analysis. Membranes were incubated overnight at 4 °C with primary antibodies—mouse anti-GFP tag antibody (Thermo Fisher Scientific, USA; 1:1000 dilution) or mouse anti-HA tag antibody(Cell Signaling Technology, Boston, MA, USA; 1:1000 dilution)—followed by incubation with horseradish peroxidase (HRP)-conjugated goat anti-mouse IgG H&L secondary antibody (Solarbio, China; 1:5000 dilution). Protein bands were detected using a chemiluminescence gel imaging system [11].

### 2.8. Construction of B. abortus ΔBPE159 and ΔBPE159-C

*BPE159* deletion strains were constructed as previously described [12]. Primers for the upstream and downstream homology arms of *BPE159* were designed (Supplementary Table S1). *BPE159* 458 bp upstream and 543 bp downstream homology arms were amplified by PCR. The kanamycin gene was amplified using Kan-F and Kan-R primers (Supplementary Table S1). The *BPE159* upstream and downstream homology arms and kanamycin gene products were subjected to a first round of fusion PCR. BPE159-N-F and BPE159-C-R (0.8 μL each) were used for second-fusion PCR. PCR products of the two rounds were extracted, cloned into a pMD19-T vector, and transformed into *Brucella* through electroporation. During screening, transformants were selected using 100 μg/mL ampicillin and 100 μg/mL kanamycin. The presence of *BPE159* was further confirmed by PCR amplification and DNA sequencing.

*B. abortus* genomic DNA was amplified and cloned into pMD19-T for generating BPE159-T. This plasmid was subcloned into pBBR1MCS4 for generating pBBR1-BPE159. This fragment was isolated based on its ampicillin-resistant phenotype and transformed into the ΔBPE159 strain through electroporation to generate a BPE159 complementary strain (ΔBPE159-C) that was screened on a TSA containing 100 mg/mL ampicillin.

### 2.9. Macrophage Infection with B. abortus S2308, ΔBPE159, and ΔBPE159-C

Two experimental groups were established: one was subjected to a standard bacterial infection, while the other was pre-incubated for 2 h with 1 µM of the lysosomal inhibitor bafilomycin A1 (Baf-A1) prior to infection. RAW264.7 cells (1.0 × 10^6^ cells/well) were cultured in 6-well plates and infected with *B. abortus* S2308, ΔBPE159, and ΔBPE159-C at a multiplicity of infection (MOI) of 100. After incubating at 37 °C under 5% CO_2_ for 60 min, the infected cells were washed thrice with sterile PBS. Subsequently, 50 mg/mL gentamicin (Sigma-Aldrich, Saint Louis, MO, USA) was added to the culture medium, followed by 50 min incubation and three PBS washes. Finally, fresh DMEM medium was introduced and the cultures were maintained at 37 °C in an incubator.

### 2.10. Construction of Growth Curve

Positive monoclonal colonies of the parental, ΔBPE159, and ΔBPE159-C strains were picked into three different vials, each containing 20 mL tryptic soy broth, and incubated in a shaker at 37 °C 180 rpm. The optical density at 600 nm (OD_600_) was initially adjusted to 0.1, and the OD_600_ values of the three groups were determined every 2 h.

### 2.11. Intracellular Survival

Positive monoclonal colonies of the parental, ΔBPE159, and ΔBPE159-C strains were picked into three different vials, each containing 20 mL *Brucella* liquid medium, and incubated at 37 °C in a shaker. RAW264.7 cells (1.0 × 10^6^ cells/well) were cultured in 6-well plates and infected with *B. abortus* S2308, ΔBPE159, and ΔBPE159-C at a multiplicity of infection (MOI) of 100. At 0, 8, 12, and 24 h post-infection, infected cells were washed thrice with sterile phosphate-buffered saline (PBS) to remove extracellular *Brucella*, and 1 mL cell lysis buffer (containing PBS and 0.1% Triton X-100) was added to each well. The lysates were diluted 100- and 1000-fold, plated on TSA (BioLife), and viable bacteria were quantified.

### 2.12. RNA Extraction and qRT-PCR

Total RNA was extracted from infected cells using TRIzol (Takara Bio, Kusatsu, Japan) and reverse transcribed into cDNA using a Prime Script real-time PCR Kit (CoWin Biotech, Taizhou, Jiangsu, China). qRT-PCR was conducted on ABI QuantStudio 5 (Thermo Fisher Scientific, USA). Gene expression was analyzed using the 2^−△△Ct^ method. Each experiment was repeated at least thrice.

### 2.13. Western Blot

Cells were harvested 24 h post-infection with either the wild-type strain S2308, ΔBPE159, or the complementary strain ΔBPE159-C. The cell pellets were lysed on ice for 30 min using 1 mL of RIPA lysis buffer (Solarbio, Beijing, China). Total protein was extracted, and its concentration was determined with a BCA assay kit (Solarbio, Beijing, China). Proteins were separated by SDS-PAGE and subsequently transferred to a 0.45-μm PVDF membrane. After blocking with 5% skim milk for 2 h, the membrane was incubated overnight at 4 °C with the following primary antibodies: anti-p62 (Proteintech, Wuhan, China; 1:20,000 dilution), anti-LC3B (Cell Signaling Technology, Boston, MA, USA; 1:1000 dilution), and anti-β-actin (Solarbio, China; 1:5000 dilution). Following primary antibody incubation, the membrane was treated with appropriate secondary antibodies for 1 h at room temperature. Protein bands were visualized using a gel imaging system, and band intensities were quantified with Image J 1.8.0_345 software. All experiments were performed in triplicate.

### 2.14. Statistical Analysis

Each experiment was repeated at least thrice, with three replicates. One- and two-way analysis of variance was performed using GraphPad Prism 5.0 software (GraphPad Software, San Diego, CA, USA), and the results are expressed as mean ± standard deviation.

## 3. Results

### 3.1. BPE159 Is Involved in Regulatory Function in the Host Nucleus

**Figure 1:**
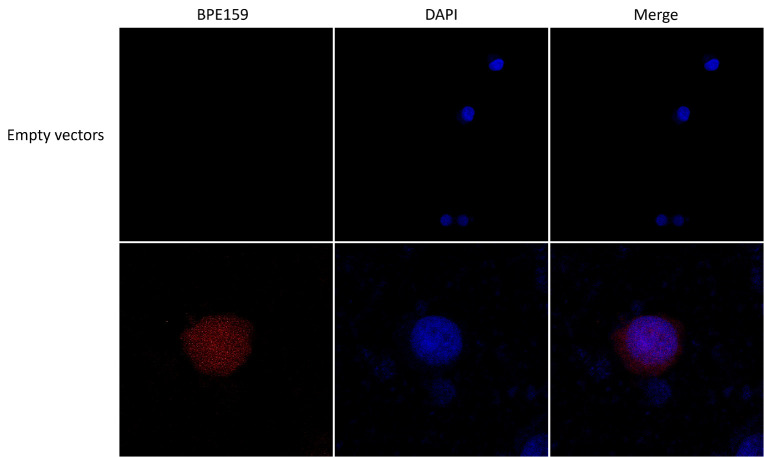
Identification of BPE159 in the nucleus of host HEK293T cells. pDsRed 2-C1-BPE159 and pDsRed 2-C1 (empty) were transferred into HEK293T cells and cultured at 37 °C, CO_2_ incubator for 24 h before confocal microscopy to observe cell morphology. The results in the photos are from three independent replicate experiments.

To determine the subcellular localization of BPE159 protein, we transfected pDsRed 2-C1-BPE159 into HEK293T cells and stained the nuclei. Confocal microscopy revealed that BPE159 was mainly located in the nucleus of host cells, and BPE159 was not found in the control group (transfected with pDsRed 2-C1 [empty]) (Figure 1). Bioinformatic analysis indicated that BPE159 did not have a signal peptide-cleavage site (Supplementary Figure S1C) or transmembrane structure (Supplementary Figure S1D); however, it had nuclear signals (Supplementary Figure S1A). These findings indicate that the protein localizes to the nucleus at the subcellular level and the BPE159 is translated within the nucleus of host cells.

### 3.2. Screening for the Interaction of BPE159 with HEK293T Cell Effector Protein

We used yeast two-hybrid technology to search for proteins that may interact with BPE159 in the RAW264.7 cDNA library. pGBKT7-BPE159 was transformed into yeast competent cells and grown on SD/-Trp, SD/-Trp/-His/X-α-gal, or SD/-Trp/-His/-AbA/X-α-gal plates. The number of white spots on SD/-Trp plates was consistent with that of the positive control and sterile spots on SD/-Trp/-His/-AbA/X-α-gal plates (Supplementary Figure S2), indicating that BPE159 is non-toxic and has no self-activating potential.

**Figure 2:**
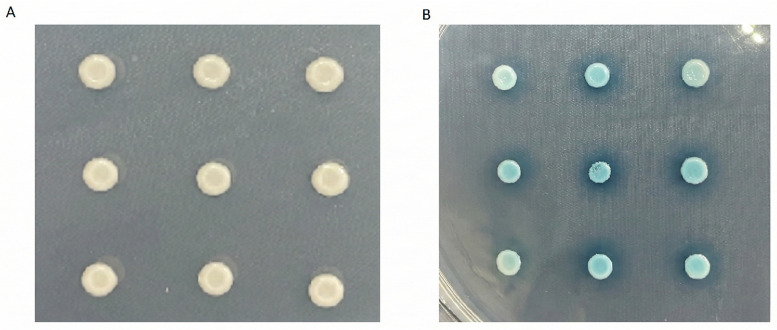
BPE159 interacts with Eif3g, Mrpl19, Eci1. (**A**) Growth results in SD/-Trp/-Leu/-Ade/-His (QDO) medium. (**B**) Growth results on SD/-Ade/-His/-Leu/-Trp/X-α-gal (QDO/X) medium. The Prey plasmid was co-transferred with the Bait plasmid into yeast competent cells, cultured in two auxotrophic plates for 5–14 days, and photographed. The results in the graph are obtained from three independent replicates.

The RAW264.7 cDNA library was transformed into yeast competent cells containing the bait plasmid pGBKT7-BPE159 and cultured. After mating and two-hybrid library screening and BLAST-positive clone sequencing, we identified three proteins that interacted with BPE159 (Table 1). Next, the Prey plasmid was co-transformed with the Bait plasmid into yeast Y2HGold competent cells, and positive monoclonal cells were screened using two auxotrophic plates, SD/-Trp/-Leu/-Ade/-His and SD/-Trp/-Leu/-Ade/-His/X-α-gal/AbA. All three proteins individually interacted with BPE159 (Figure 2).

### 3.3. Verification of the Possible Interaction Between Yeast Double Hybridization Screening and True Interaction with BPE159 Protein

**Figure 3:**
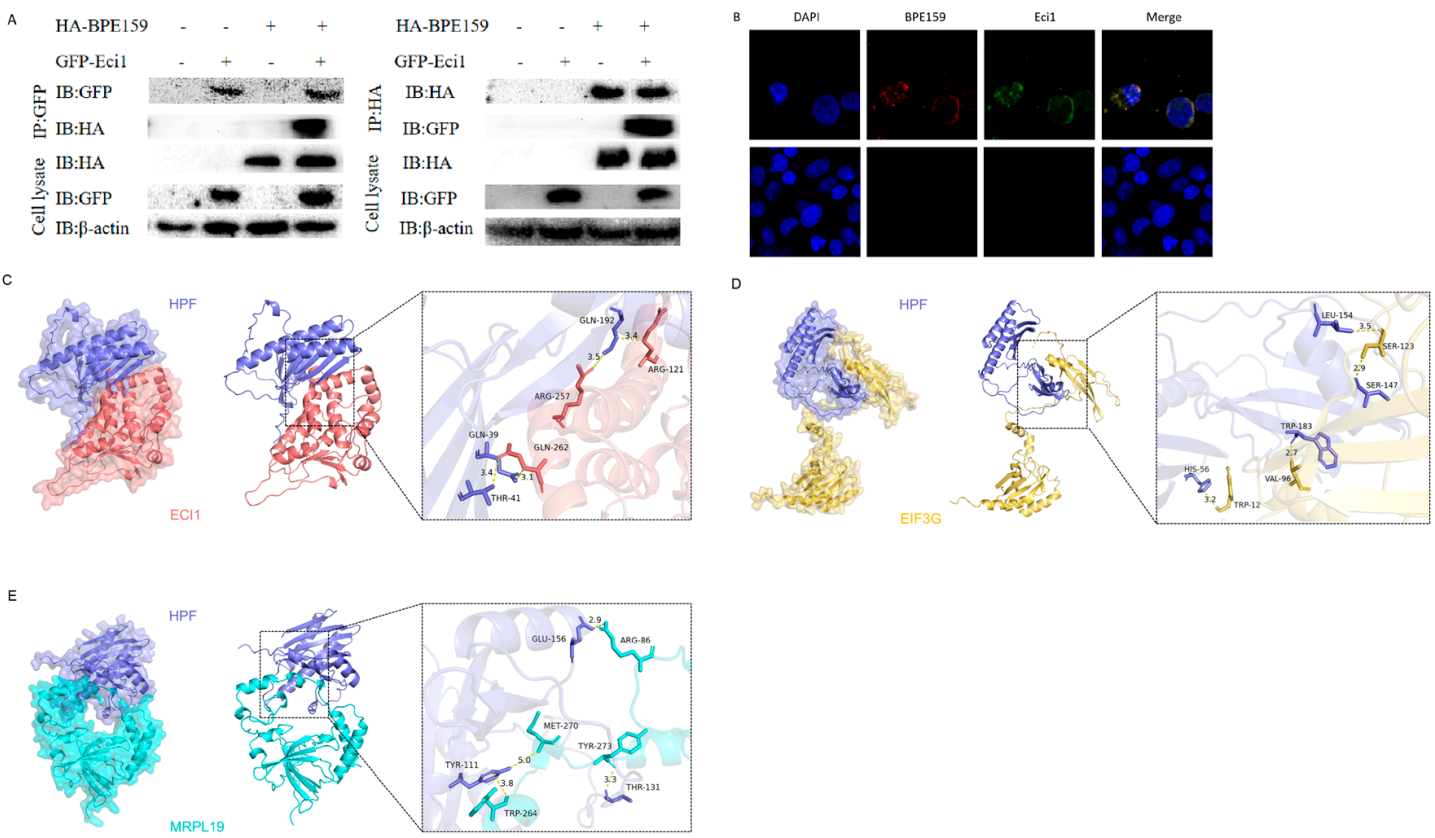
Direct interaction between BPE159 and Eci1. (**A**) BPE159 interacts with Eci1. HA-BPE159, GFP-Eci1, and empty vectors were transfected into HEK 293T cells, and the results were used alone or in combination. After 48 h of single or co-transfection, cells were harvested and incubated with anti-GFP antibody (**left**) or anti-HA antibody (**right**) for co-immunoprecipitation. Immunoblotting was performed with the indicated antibody. (**B**) Laser confocal microscopy detected the localization of BPE159 and Eci1 in HEK 293T host cells. pCDNA3.1-EGFP-Eci1 and pDsRed 2-C1-BPE159 were co-transfected into HEK293T cells, and the cells were harvested 24 h after transfection and observed by confocal microscopy. Spatial docking of (**C**) BPE159 and Eci1 proteins, (**D**) BPE159 and Eif3g proteins, and (**E**) BPE159 and Mrpl19 proteins was mapped using the Easy2MD online platform.

Yeast two-hybrid screening identified Mrpl19, Eif3g, and Eci1 that interacted with BPE159. Molecular docking also indicated that BPE159 interacted with Eci1, Eif3g, and Mrpl19 (Figure 3C–E). Next, coimmunoprecipitation was used to verify the interaction between these proteins and BPE159. We transfected both pAcGFP1-C-Prey and pCMV-HA-BPE159 into HEK293T cells and performed Western blot analysis using whole-cell lysate, both of which detected proteins. However, immunoprecipitation showed the presence of only Eci1 (Figure 3A), indicating an interaction between Eci1 and BPE159. Next, we co-transfected pDsRed2-C1-BPE159 and pCDNA3.1-EGFP-Eci1 into HEK293T cells; confocal microscopic analysis showed colocalization of BPE159 and Eci1 (Figure 3B).

### 3.4. GO Analysis of BPE159-Interacting Proteins

**Figure 4:**
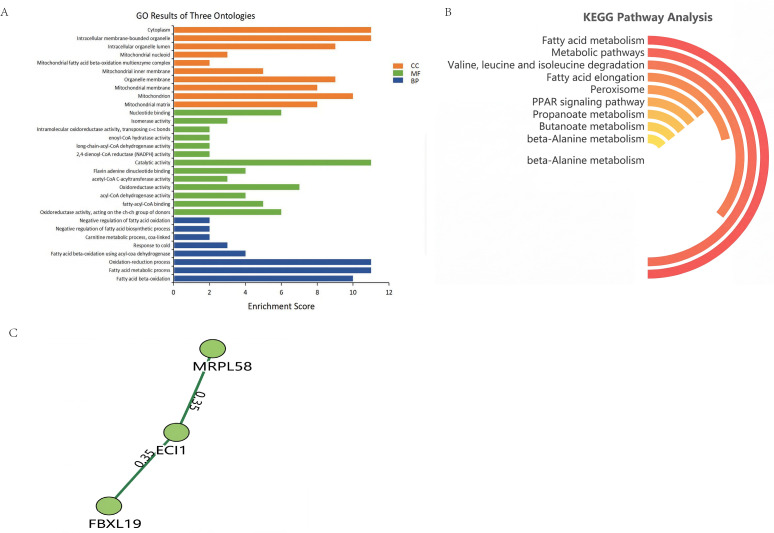
Eci1 GO terminology analysis, pathway analysis, and PPI analysis. BP, biological processes; CC, cellular component; MF, molecular function. (**A**) Eci1 protein GO terminology analysis. (**B**) Analysis of the KEGG pathway of the Eci1 protein. (**C**) PPI analysis of Eci1 protein.

To investigate the biological processes associated with Eci1, we conducted GO enrichment analysis (Figure 4A). Eci1 mainly played roles in mitochondria, intracellular organelles, and chromatin. It had catalytic and oxidative activities and was involved in nucleotide sequence linkages. KEGG analysis showed that Eci1 was mainly involved in cellular metabolic pathways such as fatty acid metabolism; metabolism and degradation of valine, leucine, and isoleucine; and signal transduction in the PPAR signaling pathway (Figure 4B). Next, we predicted potential proteins that might interact with the Eci1 and identified two such proteins—MRPL58 and FBXL19 (Figure 4C).

### 3.5. BPE159 Promotes Intracellular Survival of Brucella by Inhibiting Eci1 Expression to Reduce the Expression of Macrophage Autophagy Factors

**Figure 5:**
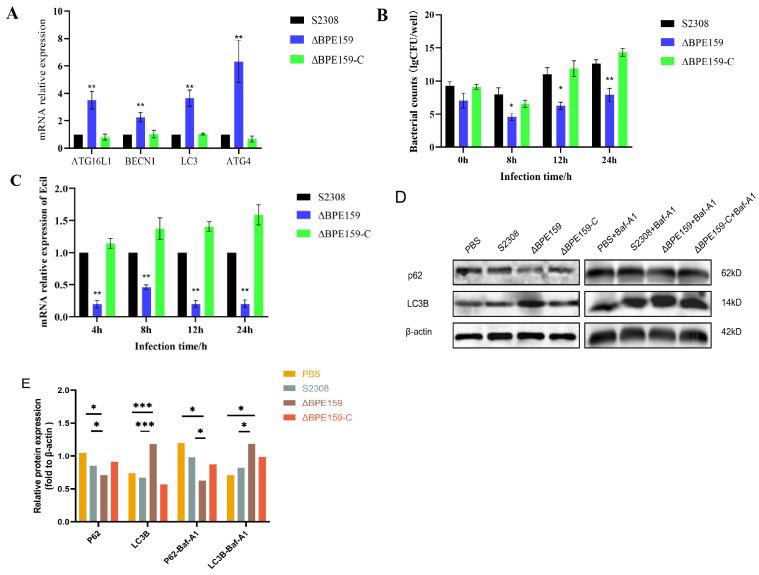
BPE159 enhances *Brucella* cellular viability and inhibits apoptosis in macrophages and promotes the expression of the interacting protein Eci1. (**A**) BPE159 enhances the apoptosis efficiency of macrophage RAW264.7. The parent, deletion, and complement strains were infected with RAW264.7 cells. After 24 h of infection, real-time fluorescence quantification was performed. All data are shown as the average from three independent tests ± standard deviation, * *p* < 0.05, ** *p* < 0.01. (**B**) BPE159 enhances cell survival of *Brucella*. Colony forming unit counting of intracellular bacteria was performed at 0, 8, 12, and 24 h post-infection. (**C**) The loss of BPE159 reduced the expression of Eci1. After infection with *Brucella* ΔBPE159 and its parent strains, cell samples were collected at 4, 8, 12, and 24 h to detect the transcription of ECI1. (**D**) The deletion of BPE159 enhances autophagy. Western blot (WB) analysis was used to detect the levels of autophagy-related proteins (p62, LC3B) in RAW264.7 cells 24 h after infection with *B. abortus*, ΔBPE159, and ΔBPE159-C. (**E**) Quantitative analysis of WB results indicate that the BPE159 protein inhibits autophagy, thereby promoting the intracellular survival of *Brucella* (* *p* < 0.05, *** *p* < 0.001). The results presented are of three independent tests. The data are shown as average ± standard deviation; * *p* < 0.05, *** *p* < 0.001.

Next, we sought to determine whether BPE159 affects Eci1 expression. BPE159 enhanced *Eci1* mRNA expression in RAW264.7 cells infected with *Brucella* (Figure 5C), and no significant difference in expression was noted between 4 and 24 h (Figure 5C), indicating that this enhancement was not time-dependent.

Next, we infected macrophages with *Brucella* S2308, S2308-ΔBPE159, and S2308-ΔBPE159-C; the survival capacity of cells varied among different bacterial strains (Figure 5B). The ΔBPE159-C strain exhibited similar survival to the parental strain, with no significant difference (*p* > 0.05); compared with the parental strain, the ΔBPE159 strain showed a reduced overall survival trend. The survival rate of *Brucella* in RAW264.7 cells was significantly lower than that of the parental strain (Figure 5B). These findings indicate that BPE159 deletion impairs the intracellular survival of *Brucella*, suggesting its potential role as a virulence factor.

S2308-ΔBPE159 and its parental strain were infected into RAW264.7 cells, and autophagy transcription and expression were detected after 24 h. Fluorescence quantification results (Figure 5A) indicated that the autophagy factors ATG16L1, BECN1, ATG4, and LC3 were all grouped after *BPE159* deletion compared with that in the parental strains (*p* < 0.05, respectively), and no significant changes in the expression of autophagy factors were noted in the complement strain (*p* > 0.05). Western blot analysis (Figure 5D,E) demonstrated that in both the normal and autophagy inhibitor groups, the deletion of BPE159 protein significantly enhanced autophagy levels 24 h post-infection, compared with those in the control group (*p* < 0.05, *p* < 0.001), indicating that BPE159 protein promotes the intracellular survival of *Brucella* by inhibiting autophagy.

## 4. Discussion

*Brucella* parasitizes host cells for a long time and does not possess typical virulence factors such as capsules, exotoxins, and cytolysins [13]. It relies on the T4SS to transport secreted proteins to host cells and aid in infection [2,14]. As a newly identified secretory protein, the functional dependence of BPE159 on T4SS remains to be elucidated. The deletion of *Brucella* secretory protein genes *VceA* and *VceC* does not affect the growth and reproduction of *Brucella*; however, its survival with the human trophoblast cells is decreased [15]. Our study showed a decrease in the intracellular carrier volume after *BPE159* deletion, indicating that BPE159 helps *Brucella* to evade the host immune mechanism for survival in host cells.

In our study, both bioinformatic and confocal microscopic analyses demonstrated that BPE159 operated through the host nucleus. Similar to virulence factors employed by other bacterial pathogens, it is plausible that *Brucella* effector proteins may fulfill analogous roles in mediating bacterial pathogenesis (including apoptosis, autophagy, and inflammation) [16]. Autophagy is stimulated when pathogenic bacteria invade host cells, thereby trapping and eliminating them [17]. However, host autophagy can be manipulated by pathogenic bacteria to improve their survival. During *Brucella* infection, phagocytic vesicles in host cells do not bind to lysosomes after reaching the ER; however, they are converted to autophagosomes for binding [4]. This reduces the possibility of eliminating pathogenic bacteria from host cells.

Pathogenic bacteria use autophagy proteins for regulating autophagy and achieving self-survival. The autophagy-related protein ATG4 regulates the formation of autophagic vacuoles [18]. ATG16L1 can specifically bind to pathogenic virulence factors, such as autophagy induced by *Staphylococcus aureus*, which relies on this interaction [19]. A *VirB* promoter deletion mutant of *Brucella* significantly increases the mRNA level of BECN1 in dendritic cells, suggesting that deletion of the *VirB* promoter may enhance *Brucella*-induced autophagy. In the present study, infection with wild strains of *Brucella* decreased *ATG4, ATG16L1, LC3,* and *BECN1* levels in RAW264.7 cells. Deletion of *BPE159* increased the mRNA expression of these genes in RAW264.7 cells. This finding is similar to that previously reported for Mouse macrophages.

**Figure 6:**
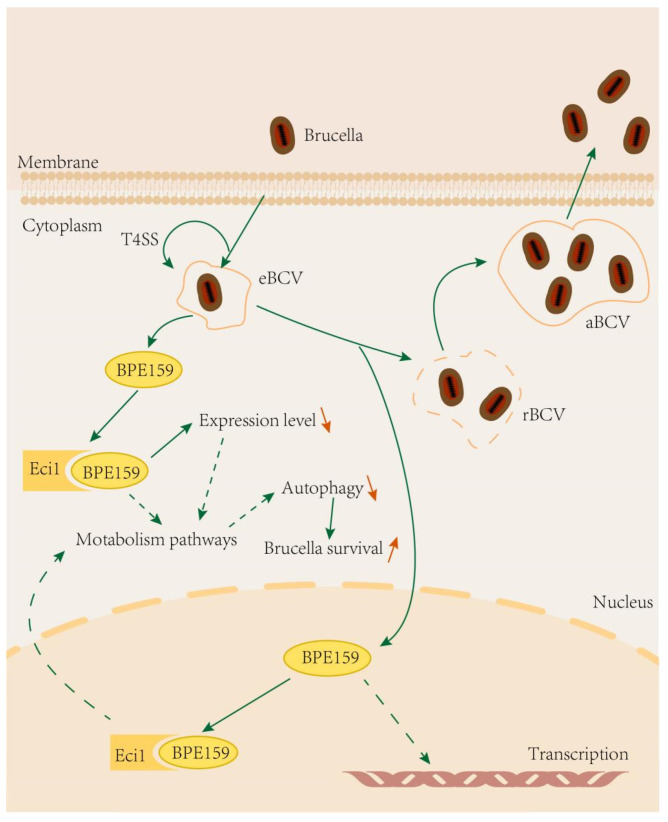
Model of *Brucella abortus* effector protein BPE159 enhances bacterial intracellular survival targeting the host autophagy regulator Eci1 by inhibiting autophagic flux. The solid arrow corresponds to the established findings, while the dashed arrow indicates the potential results.

*Eci1* encodes a member of the hydrase/isomerase superfamily and a protein that is a key mitochondrial enzyme involved in β-oxidation of unsaturated fatty acids. *Eci1* catalyzes stepwise degradation of cis, mono, and polyunsaturated fatty acids into 3-cis- and 3-trans-ale-elate intermediates, respectively, produced during the process of 2-trans-enoyl-CoA formation. This is an important step in the β-oxidation of unsaturated fatty acids. *Eci1* overexpression increases the growth of prostate cancer (PCa) cells *in vitro*, whereas its suppression reduces PCa cell growth [20]. Additionally, ECI1 overexpression significantly improved the colony-forming ability, motility and maximum mitochondrial respiratory rate of PCa cells. Although mitochondrial fatty acid β-oxidation disorder has been shown to play a key role in the pathogenesis of PCa, the biological function of *Eci1* in brucella infection remains poorly studied (Figure 6).

Upon host cell entry, BPE159 specifically targets the host factor Eci1, functioning as a negative regulator of this downstream gene, thereby attenuating autophagic processes and enhancing bacterial persistence.

## 5. Conclusions

The findings of this study indicate that BPE159 may regulate autophagy in host cells by interfering with cellular metabolic pathways, thereby enabling *Brucella* survival within host cells. However, the further exploration of mechanistic details is warranted.

## Figures and Tables

**Table 1:** Analysis of three potential interacting proteins with BPE159 protein.

Number	BLAST Comparison Prediction Results	NCBI Number	Length
Eci1	Enoyl-CoQ Aδ isomerase 1, mitochondrial precursor	NP_034153.2	12,634 bp
Eif3g	Eukaryotic translation initiation factor 3 subunit G	NP_058572.2	4242 bp
Mrpl19	39S ribosomal protein L19	NP_080766.1	8124 bp

## Data Availability

The data presented in this study are available at https://www.ncbi.nlm.nih.gov/ (accessed on 1 November 2025).

## Supplementary Materials

The following supporting information can be downloaded at: https://www.mdpi.com/article/10.3390/microorganisms14030663/s1, Table S1: Primers were used to construct the BPE159 deletion strain and complementation strains and construction of fluorescent co-localization vectors. Additionally, they were used to perform quantitative real time–polymerase chain reaction (qRT–PCR); Figure S1: Bioinformatics analysis of BPE159; Figure S2: Results of *BPE159* self-activation and toxicity tests were reported.

## Abbreviations

The following abbreviations are used in this manuscript:

BPE	*Brucella* putative effector
T4SS	type IV secretion system
BCV	*Brucella*-containing vacuole
eBCVs	endosomal *Brucella*-containing vacuoles
rBCVs	replicative *Brucella*-containing vacuoles
ER	endoplasmic reticulum
TSA	tryptic soy agar
qRT-PCR	quantitative reverse-transcription polymerase chain reaction
DDO	SD/–Leu/–Trp
QDO	SD/–Ade/–His/–Leu/–Trp
QDO/X	SD/–Ade/–His/–Leu/–Trp/X-α-gal
Baf-A1	bafilomycin A1
PBS	phosphate-buffered saline
MOI	multiplicity of infection
SD	synthetic dropout
GO	Gene Ontology
KEGG	Kyoto Encyclopedia of Genes and Genomes
PCa	prostate cancer
ΔBPE159	BPE159-deleted
ΔBPE159-C	BPE159-complementary
