# Supplementary material for: Nuclear Localization of Effector BPE159: A Pivotal Mechanism for Intracellular Persistence of Brucella by Hampering Host Autophagy
Source: Microorganisms. 2026 Mar 14;14(3):663. doi: 10.3390/microorganisms14030663 (PMC13029306; doi:10.3390/microorganisms14030663)
Supplement: Supplementary file 1 [file microorganisms-14-00663-s001.zip › microorganisms-4109950-supplementary.pdf]

SUPPLEMENTARY INFORMATION

Nuclear Localization of Effector BPE159: A Pivotal Mechanism for Intracellular Persistence of Brucella by Hampering Host Autophagy

Yidan Zhang [1,#],Tingting Lyu[1,#],Shengnan Song[1],Yu Zhang[1] ,Chunyan Wei[1] ,Liangbo Liu[1] ,Zhen Wang[1] ,Zhihua Sun[1] ,Xia Zhou[1] ,Jia Guo[1,*] and Hui Zhang [1,*]

1 Affiliation 1; State International Joint Research Center for Animal Health Breeding, College of Animal Science and Technology, Shihezi University, Shihezi 832003, China
# These authors contributed equally to this work
* Correspondence: Jia Guo zhguojia@stu.shzu.edu.cn, Hui Zhang shz_zhang@126.com

SUPPLEMENTARY REFERENCES

Supplementary Table S1. Primers were used to construct the BPE159 deletion strain and complementation strains and construction of fluorescent co-localization vectors. Additionally, they were used to perform quantitative real time–polymerase chain reaction (qRT–PCR).

Primer name	sequence(5′→3′)
PDsRed2-C1-BPE159-F	CCCTCGAGATGACTCTGCGTGTA
PDsRed2-C1-BPE159-R	CGGAATTCTCAGGCGCGTGG
pGBKT7-BPE159-F	CGGAATTCTCAGGCGCGTGG
pGBKT7-BPE159-R	AACTGCAGTCAGGCGTGG
pCMV-HA-BPE159-F	CTCTAGACATGACTCTGCGTGTATC
pCMV-HA-BPE159-R	TATAGAATTCATATCAGGCGCGTG
pCDNA3.1-EGFP-Eci1-F	TCCATATGAGGCGCTGGCTGCTGC
pCDNA3.1-EGFP-Eci1-R	CCCAAGCTTTTAGCCCTTCTTTTGCTTGAGCTTTTCCAAG
pAcGFP1-C-Eci1-F	CGCGTCGACATGGCGCTGGCTGCTGC
pAcGFP1-C-Eci1-R	GCTCTAGATTAGCCCTTCTTTTGCTTGAGCTTTTCCAAGT
BPE159-N-F	ATCTGTGGAAACAAGGGCG
BPE159-N-R	GACATTCATCCCAGGTGGCTGGTCCACCTCATTTGCGT
BPE159-C-F	TCTGGGGTTCGAAATGACCGTGGCAAACCAGAAGCGC
BPE159-C-R	AGCCGTCAGGAATGAATAGAGG
Kan-F	GCCACCTGGGATGAATGTC
Kan-R	CGGTCATTTCGAACCCCAGA
BPE159-F	CCCTCGAGATGACTCTGCGTGTA
BPE159-R	CGGAATTCTCAGGCGCGTGG
GAPDH-F	CAGCCTCAAGATCATCAGCA
GAPDH-R	TGTGGTCATGAGTCCTTCCA
ATG16L1-F	ATGTCGTCGGGCCTGC
ATG16L1-R	TCAAGGCTGTGCCCACAGCACA
BECN1-F	ATGATGTCTACAGAAAGTGCTAATAGCT
BECN1-R	TCACTTGTTATAGAACTGTGAGGACAC
LC3-F	ATGCCCTCCGACCGGCCTTTCAA
LC3-R	TCAGAAGCCGAAGGTTTCTTGGG
ATG4-F	ATGGACGCAGCCACTTTGACA
ATG4-R	TCAGAGGGATAAGATTTCAAAGTC

Supplementary FIGURE S1. Bioinformatics analysis of BPE159.

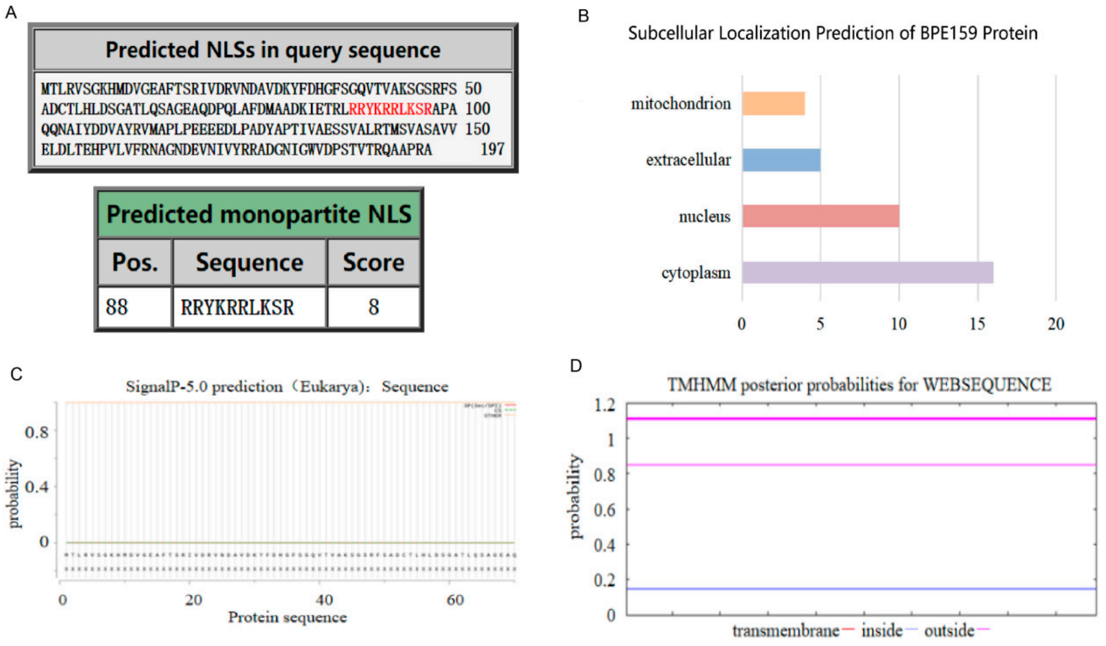

(A)Predicted by BPE159 Nuclear Localization Signal (NLS).(B)Results of BPE159 subcellular localization prediction.(C)Predict the amino acid sequence of BPE159 protein using SignalP5.0 Server.(D)The transmembrane domain of BPE159 protein was predicted by TMHMM Server analysis.

Supplementary FIGURE S2. Results of *BPE159* self-activation and toxicity tests were reported.

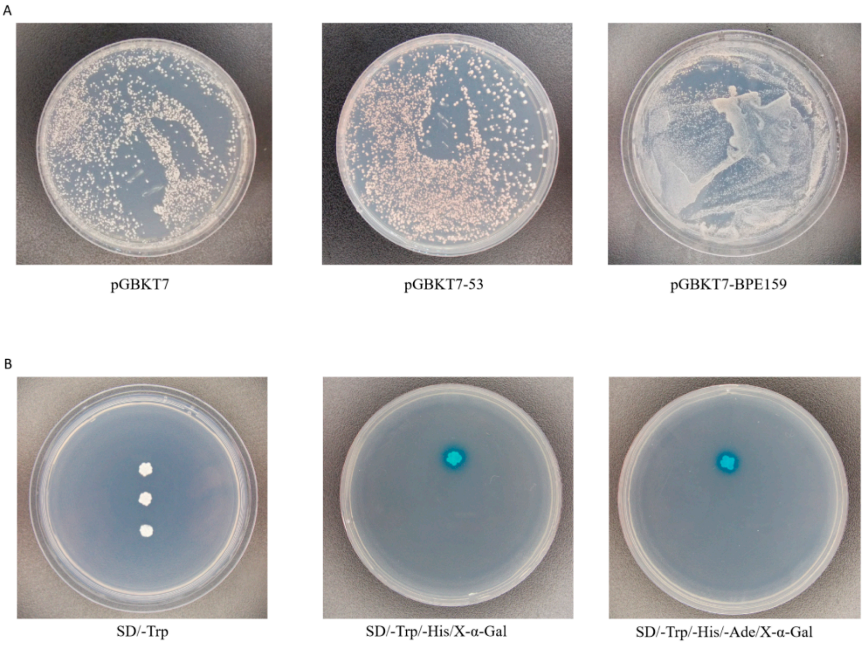

(A)*BPE159* self-activation assay.(B)*BPE159* toxicity assay.
